# Improved prognostic stratification of patients with isocitrate dehydrogenase-mutant astrocytoma

**DOI:** 10.1007/s00401-023-02662-1

**Published:** 2024-01-06

**Authors:** Michael Weller, Jörg Felsberg, Bettina Hentschel, Dorothee Gramatzki, Nadezhda Kubon, Marietta Wolter, Matthias Reusche, Patrick Roth, Dietmar Krex, Ulrich Herrlinger, Manfred Westphal, Joerg C. Tonn, Luca Regli, Claude-Alain Maurage, Andreas von Deimling, Torsten Pietsch, Emilie Le Rhun, Guido Reifenberger

**Affiliations:** 1 Department of Neurology, University Hospital Zurich, Frauenklinikstrasse 26, 8091 Zurich, Switzerland; 2 Department of Neurology, University of Zurich, Zurich, Switzerland; 3 Institute of Neuropathology, Heinrich Heine University, Medical Faculty, and University Hospital Düsseldorf, Düsseldorf, Germany; 4 Institute for Medical Informatics, Statistics and Epidemiology, University Leipzig, Leipzig, Germany; 5 Faculty of Medicine, Department of Neurosurgery, Technische Universität Dresden, University Hospital Carl Gustav Carus, Dresden, Germany; 6 Department of Neurology, University of Bonn, Bonn, Germany; 7 Department of Neurosurgery, University of Hamburg, Hamburg, Germany; 8 Department of Neurosurgery, Ludwig-Maximilians-University Munich, Munich, Germany; 9 German Cancer Consortium (DKTK), Partner Site Munich, Munich, Germany; 10 Department of Neurosurgery, University Hospital Zurich, Zurich, Switzerland; 11 Department of Neurosurgery, University of Zurich, Zurich, Switzerland; 12 Department of Pathology, Centre Biologie Pathologie, Lille University Hospital, Hopital Nord, Lille, France; 13 Department of Neuropathology, University Hospital Heidelberg, Heidelberg, Germany; 14 Clinical Cooperation Unit Neuropathology, German Cancer Center (DKFZ), and German Cancer Consortium (DKTK), Partner Site Heidelberg, Heidelberg, Germany; 15 Department of Neuropathology, University of Bonn Medical Center, DGNN Brain Tumor Reference Center, Bonn, Germany; 16 Department of Neurosurgery, Lille University Hospital, Lille, France; 17 German Cancer Consortium (DKTK), Partner Site Essen/Düsseldorf, Düsseldorf, Germany

**Keywords:** Brain, *CDKN2A*, CNS WHO grade, IDH, *LINE-1*, Molecular

## Abstract

Prognostic factors and standards of care for astrocytoma, isocitrate dehydrogenase (IDH)-mutant, CNS WHO grade 4, remain poorly defined. Here we sought to explore disease characteristics, prognostic markers, and outcome in patients with this newly defined tumor type. We determined molecular biomarkers and assembled clinical and outcome data in patients with IDH-mutant astrocytomas confirmed by central pathology review. Patients were identified in the German Glioma Network cohort study; additional cohorts of patients with CNS WHO grade 4 tumors were identified retrospectively at two sites. In total, 258 patients with IDH-mutant astrocytomas (114 CNS WHO grade 2, 73 CNS WHO grade 3, 71 CNS WHO grade 4) were studied. The median age at diagnosis was similar for all grades. Karnofsky performance status at diagnosis inversely correlated with CNS WHO grade (*p* < 0.001). Despite more intensive treatment upfront with higher grade, CNS WHO grade was strongly prognostic: median overall survival was not reached for grade 2 (median follow-up 10.4 years), 8.1 years (95% CI 5.4–10.8) for grade 3, and 4.7 years (95% CI 3.4–6.0) for grade 4. Among patients with CNS WHO grade 4 astrocytoma, median overall survival was 5.5 years (95% CI 4.3–6.7) without (*n* = 58) *versus* 1.8 years (95% CI 0–4.1) with (*n* = 12) homozygous *CDKN2A* deletion. Lower levels of global DNA methylation as detected by *LINE-1* methylation analysis were strongly associated with CNS WHO grade 4 (*p* < 0.001) and poor outcome. *MGMT* promoter methylation status was not prognostic for overall survival. Histomolecular stratification based on CNS WHO grade, *LINE-1* methylation level, and *CDKN2A* status revealed four subgroups of patients with significantly different outcomes. In conclusion, CNS WHO grade, global DNA methylation status, and *CDKN2A* homozygous deletion are prognostic in patients with IDH-mutant astrocytoma. Combination of these parameters allows for improved prediction of outcome. These data aid in designing upcoming trials using IDH inhibitors.

**Supplementary Information:**

The online version contains supplementary material available at 10.1007/s00401-023-02662-1.

## Introduction

The 2016 revision of the World Health Organization (WHO) classification of tumors of the central nervous system (CNS) had placed major emphasis on the isocitrate dehydrogenase (IDH) mutation status when classifying diffuse gliomas in adults [29]. Patients with diffuse gliomas with seemingly similar histology had very different outcomes when stratified for IDH mutation status [2–4, 8, 24, 52]. However, the diagnostic separation of adult-type diffuse astrocytic gliomas into IDH-mutant and IDH-wildtype tumors has generated new challenges regarding the role of grading and molecular prognosticators within these newly defined tumor types [7]. Detection of an IDH mutation in a diffuse astrocytic glioma with microvascular proliferation or necrosis is no longer compatible with a glioblastoma diagnosis, i.e., such tumors are now diagnosed as astrocytoma, IDH-mutant, CNS WHO grade 4 [7, 30]. Moreover, homozygous deletion of the *CDKN2A/CDKN2B* tumor suppressor gene locus has been introduced as a molecular biomarker for CNS WHO grade 4 in an IDH-mutant astrocytoma [30, 41]. Other molecular alterations that have been associated with aggressive behavior and shorter survival include high tumor mutational burden and increased copy number variation load, as well as various aberrations affecting single genes or chromosomes, such as type of IDH mutation, *PIK3R1* mutation, *PDGFRA* amplification, copy number neutral loss of 17p, loss of 19q, and others [42]. In addition, reduced global DNA methylation, referred to as glioma CpG island methylator phenotype low (gCIMPlow), has been associated with worse outcome [11, 41]. Detection of the global DNA methylation status can be accomplished by microarray- or sequencing-based methylome analyses [9, 11, 31], with focused methylation analysis of the *LINE-1* repetitive element being reported as a valuable surrogate marker for global DNA methylation level assessment [57].

Histological grading of IDH-mutant astrocytomas is subject to inter-observer variability [29] and its prognostic relevance is a matter of ongoing discussion. While some studies questioned the prognostic role of histological grading, others showed distinct outcomes according to tumor grade (for review see [7]). Further, whether patients diagnosed with IDH-mutant astrocytoma, CNS WHO grade 4, should be treated like IDH-wildtype glioblastoma patients or rather like patients with IDH-mutant astrocytoma, CNS WHO grade 3, remains controversial [47]. The IDH mutation has recently gained clinical importance as a therapeutic target since vorasidenib, an oral brain-penetrant inhibitor of mutant IDH1 and IDH2 enzymes, significantly improved progression-free survival in patients with CNS WHO grade 2 IDH-mutant gliomas [32].

To further define the prognostic roles of clinical features, CNS WHO grade, and selected molecular biomarkers in IDH-mutant astrocytoma patients, we assembled a large, clinically well documented patient cohort with long-term follow-up data from the German Glioma Network (GGN) and two institutional cohorts.

## Patients and methods

### Patients

Patients were enrolled in the GGN (*n* = 212) or followed at the University Hospitals of Lille, France (*n* = 32) or Zurich, Switzerland (*n* = 14). The GGN is a prospective, non-interventional cohort study that comprised eight University Hospitals in Germany. All GGN patients gave written informed consent for participation in the GGN and its translational research projects. Local ethics approvals were in place in Lille and Zurich. Patient characteristics, treatment, and outcome data were collected prospectively within the GGN and assembled retrospectively following a similar data capture scheme for patients from Lille and Zurich.

### Central neuropathology review

Representative tumor specimens from all patients were subjected to central pathology review at the Brain Tumor Reference Center of the German Society for Neuropathology and Neuroanatomy (DGNN) in Bonn (TP) and Düsseldorf (GR). In addition to histological confirmation of a diffuse astrocytic glioma, the tumors were histologically graded according to the World Health Organization (WHO) classification of central nervous system (CNS) tumors [30]. Accordingly, CNS WHO grade 3 tumors were distinguished from CNS WHO grade 2 tumors by the presence of focal or dispersed anaplasia and significant mitotic activity, while CNS WHO grade 4 tumors were distinguished from the CNS WHO grade 2 and 3 tumors by the presence of microvascular proliferation and/or necrosis and/or homozygous *CDKN2A/CDKN2B* deletion [30]. All tumors were screened for the IDH1-R132H mutation using immunohistochemistry with a mutation-specific monoclonal antibody (clone H09, Dianova, Hamburg, Germany) [10]. Tumors negative for IDH1-R132H by immunohistochemistry were assessed for non-canonical *IDH1* or *IDH2* mutations using Sanger sequencing or pyrosequencing [15, 21, 22]. For the molecular analyses, DNA was extracted from frozen tissue samples using the PureLink™ Genomic DNA Mini Kit (Life Technologies, Carlsbad, CA) or ultracentrifugation [23]. Alternatively, DNA was extracted from formalin-fixed and paraffin-embedded tissue samples using the QIAamp DNA FFPE Tissue Kit (Qiagen, Hilden, Germany), the GeneRead DNA FFPE Kit (Qiagen), or the Maxwell® RSC FFPE Plus DNA Kit together with the Maxwell® RSC instrument (Promega, Mannheim, Germany). Tumor tissue samples used for DNA extraction were histologically evaluated to contain a sufficient tumor cell content of usually more than 80%. In four tumors (1 CNS WHO grade 2 and 3 CNS WHO grade 4 tumors), classification as IDH-mutant astrocytoma was based only on array-based DNA methylome analysis using the Heidelberg classifier version v.12.5 (https://www.molecularneuropathology.org/mnp/) without further specification of the specific *IDH1* or *IDH2* mutation by DNA sequencing (Table 1).

**Table 1:** Patient and disease characteristics by CNS WHO grade

	Astrocytoma, IDH-mutant, CNS WHO grade 2 *n* = 114	Astrocytoma, IDH-mutant, CNS WHO grade 3 *n* = 73	Astrocytoma, IDH-mutant, CNS WHO grade 4 *n* = 71
Age at diagnosis			
Median (years)	36	39	37
Range (years)	19–69	21–80	23–79
Sex			
Male	78 (68.4%)	46 (63.0%)	47 (66.2%)
Female	36 (31.6%)	27 (37.0%)	24 (33.8%)
KPS at diagnosis			
90–100	71 (86.6%)	39 (70.9%)	33 (47.1%)
70–80	11 (13.4%)	14 (25.5%)	25 (35.7%)
<70	0	2 (3.6%)	12 (17.1%)
No data	32	18	1
Tumor location			
Frontal	41 (36.0%)	27 (37.0%)	36 (50.7%)
Temporal	25 (21.9%)	16 (21.9%)	5 (7.0%)
Parietal	9 (7.9%)	4 (5.5%)	7 (9.9%)
Cerebellar, brain stem	0	0	2 (2.8%)
Not localized to one site	25 (21.9%)	18 (24.7%)	20 (28.2%)
Multifocal	1 (0.9%)	0	0
Others	12 (10.5%)	8 (11.0%)	1 (1.4%)
Unknown	1 (0.9%)	0	0
IDH mutation status			
IDH1^R132H^ mutation	101 (88.6%)	66 (90.4%)	62 (87.3%)
Other IDH1 mutations	11 (9.6%)^a^	6 (8.2%)^b^	5 (7.0%)^c^
IDH2^R172K^ mutation	1 (0.9%)	1 (1.4%)	1 (1.4%)
IDH mutation type not determined^d^	1 (0.9%)	0	3 (4.2%)
*MGMT* promoter methylation status			
Methylated	72 (65.5%)	58 (84.1%)	53 (74.6%)
Unmethylated	38 (34.5%)	11 (15.9%)	18 (25.4%)
No data	4	4	0
*CDKN2A* deletion status			
Homozygous deletion	0	0	12 (17.1%)
No homozygous deletion	66 (100%)	61 (100%)	58 (82.9%)
No data	48	12	1
*LINE-1* methylation status			
Methylated alleles (%, median)	83.0	82.7	72.8
Methylated alleles (%, range)	74.2–86.0	71.2–86.3	60.0–79.1
No data	62	15	1

*CI* confidence interval, *KPS* Karnofsky performance status

^a^Other IDH1 mutations are R132C (*n* = 4), R132G (*n* = 3), R132L (*n* = 2), R132S (*n* = 2)

^b^Other IDH1 mutations are R132C (*n* = 3), R132G (*n* = 2), R132L (*n* = 1)

^c^Other IDH1 mutations are R132C (*n* = 2), R132G (*n* = 1), R132S (*n* = 2)

^d^Assignment to methylation class astrocytoma, IDH-mutant based on DNA methylome analysis

In addition to IDH mutation testing, tumors were investigated for 1p/19q codeletion status using either microsatellite-based loss of heterozygosity (LOH) analysis [13, 58] or comparative genomic hybridization [51]. In individual cases, the 1p/19q codeletion status was determined based on copy number profiles obtained by array-based DNA methylome analysis. None of the 258 tumors demonstrated a 1p/19q codeletion.

The *MGMT* promoter methylation status was determined by methylation-specific PCR or DNA pyrosequencing [14, 46] or, in individual cases, by DNA methylome analysis using the STP27 algorithm [1]. A total of 173 of the 212 GGN cases had been included in previous GGN studies [5, 6, 19, 20, 22, 27, 36, 37, 39, 40, 48–51, 53].

### Detection of homozygous deletion of *CDKN2A* by droplet digital PCR (ddPCR)

We used a commercially available ddPCR assay (Bio-Rad Laboratories) for the detection of *CDKN2A* homozygous deletions on 9p21 [55]. The loci *NCKAP5* and *KCNS3* (2p24.2) served as reference loci. The threshold for detection of a homozygous deletion was set to a calculated relative *CDKN2A* copy number value of <0.5 which was experimentally demonstrated to reliably detect a homozygous deletion when the tumor cell content in the tissue sample used for DNA extraction was ≥75% [55].

### Determination of *LINE-1* methylation by pyrosequencing

As a surrogate marker for the global DNA methylation status [57], we determined the methylation level of the *LINE-1* repetitive element (GenBank accession number X58075) in the tumor DNA using DNA pyrosequencing of sodium bisulfite converted DNA. The primer pair LINE-1-bisu-F1 5ʹ- taggattttttgagttaggtgtg and LINE-1-bisu-R1 5ʹ-[Btn]caaaaaatcaaaaaattccctttcc (biotinylated at the 5ʹ -end) was used for amplification of a 156-bp fragment. Pyrosequencing on the PyroMark Q24 (Qiagen, Hilden, Germany) was performed using the sequencing primer LINE-1-bisu-Seq1 5ʹ -ttaggtgtgggatatagt with the sequence to analyze being “TTYGTGGTGYGTYGTTT”. The three investigated CpG sites correspond to the first three CpGs covered by the PyroMark Q96 CpG LINE-1 kit from Qiagen. After pyrosequencing, we calculated the mean value of the methylated allele percentages at the three investigated CpG sites. A ROC analysis was performed to determine an appropriate cut-off value for the *LINE-1* methylation levels, i.e., percentage of methylated alleles that distinguished best between CNS WHO grade 4 tumors as opposed to CNS WHO grade 2 or 3 tumors. Thereby, a cut-off value of ≤77% methylated alleles was calculated with an area under the curve (AUC) of 0.98.

### Array-based DNA methylation analyses

Large-scale DNA methylation data obtained by hybridization of tumor DNA to 450 k methylation bead arrays (Illumina, San Diego, CA) were available for 85 patients with IDH-mutant astrocytoma included in this study, comprising 31 CNS WHO grade 2, 31 CNS WHO grade 3, and 23 CNS WHO grade 4 tumors. *LINE-1* methylation data were available from 80 of these tumors (29 grade 2, 28 grade 3, and 23 grade 4). 450 k DNA methylation data were generated as described [9] and analyzed with the Heidelberg classifier algorithm version v.12.5 (www.molecularneuropathology.org). Tumors were assigned to the methylation classes “astrocytoma, IDH-mutant, lower grade” or “astrocytoma, IDH-mutant, high-grade” based on calibrated classifier scores of ≥0.9. Principles of the DNA methylation-based classification of central nervous system tumors, the distinction of methylation classes, and the role of calibrated classifier scores have been reported [9].

### Statistical analyses

Progression-free survival (PFS) was calculated from the day of first surgery until tumor progression, death, or end of follow-up. Overall survival (OS) was calculated from the day of first surgery until death or end of follow-up. Kaplan–Meier survival curves, Log-rank test, and Cox regression were used for univariate and multivariate analyses of survival. Chi^2^-test and Fisher’s exact test were used to analyze categorical data. Quantitative data were analyzed by *t* test and Mann–Whitney *U* test. A ROC analysis was performed to determine an appropriate cut-off value for the percentage of *LINE-1* methylated alleles. Sensitivity and specificity with 95% confidence interval (CI) were calculated.

## Results

### Patient characteristics

The median age was below 40 years for all CNS WHO grades. Less than half (47.1%) of the patients with CNS WHO grade 4 tumors had a KPS 90 or 100, as opposed to 86.6 and 70.9% of the patients with CNS WHO grade 2 or 3 tumors (*p* < 0.001). IDH-mutant astrocytomas of CNS WHO grade 4 were numerically more often located in the frontal lobes (*p* = 0.118) (Table 1), and a gross total resection was numerically more often performed in these patients than in patients with CNS WHO grade 2 or 3 tumors (*p* = 0.177) (Table S1).

### Molecular characteristics

The canonical IDH-R132H mutation was detected in almost 90% of the tumors with equal frequencies across grades. *MGMT* promoter methylation was detected in more than 70% of all tumors, with the lowest percentage of 65.5% detected in CNS WHO grade 2 tumors (Table 1). *CDKN2A* homozygous deletions were detected in 12 of 71 patients (16.9%) with CNS WHO grade 4 tumors. Eleven of these tumors also showed histological features of CNS WHO grade 4, i.e., microvascular proliferation or necrosis or both. The *LINE-1* methylation levels were lower in CNS WHO grade 4 tumors than in CNS WHO grade 2 or 3 groups (Table 1).

### Treatment and outcome

**Fig. 1:**
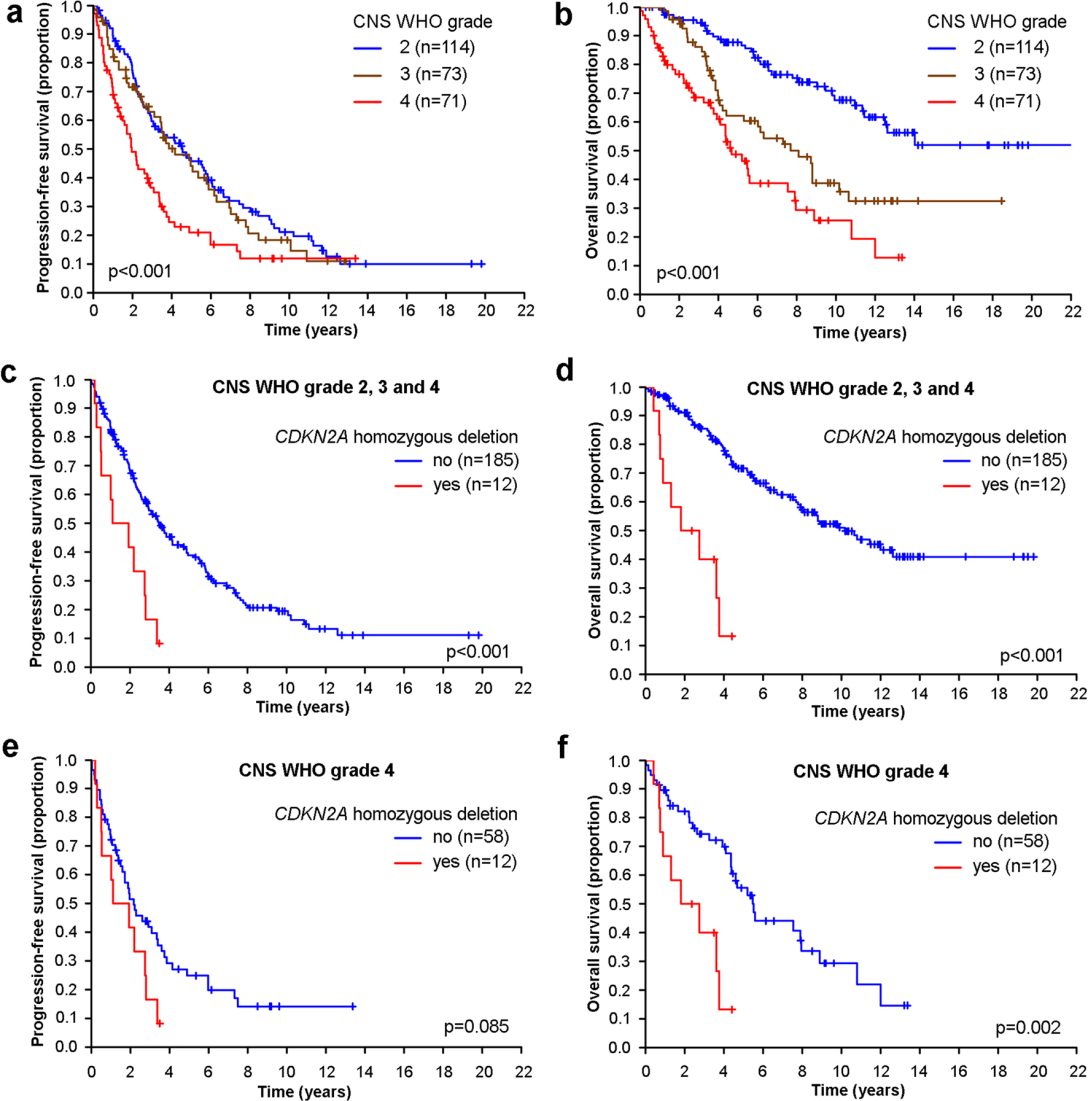
Outcome of patients with IDH-mutant astrocytoma stratified according to CNS WHO grade and *CDKN2A* copy number status. (**a**, **b**) PFS (a) and OS (b) of the entire cohort of patients with IDH-mutant astrocytomas stratified by CNS WHO grade 2, 3 or 4. (**c**–**f**) PFS (**c**, **e**) and OS (**d**, **f**) of the entire cohort of patients with IDH-mutant astrocytoma (**c**, **d**) or IDH-mutant astrocytoma of CNS WHO grade 4 only (**e**, **f**)

Patients with CNS WHO grade 4 tumors received combined modality treatment upfront more often than patients with CNS WHO grade 2 or 3 tumors. A wait-and-scan strategy was more commonly adopted in patients with CNS WHO grade 2 tumors than in patients with CNS WHO grade 3 or 4 tumors. PFS did not differ between patients with CNS WHO grade 2 versus 3 tumors (*p* = 0.557), but was significantly lower in patients with CNS WHO grade 4 tumors (*p* < 0.001) (Fig. 1a, Table S1). At progression, 36 of 50 CNS WHO grade 2 tumors (72%) and 4 of 15 CNS WHO grade 3 tumors (27%) that were subjected to repeated surgery showed histological progression to a higher grade in the recurrent tumor tissue. The rates of documented progression events were similar across the tumor grades. The percentages of patients treated at first progression varied from 90.5% in patients with CNS WHO grade 2 tumors to 81.6 and 82.1% for patients with CNS WHO grade 3 and 4 tumors.

Survival was longer for patients with CNS WHO grade 2 tumors than for patients with CNS WHO grade 3 or 4 tumors (*p* < 0.001) (Table S1, Fig. 1b). Furthermore, patients with CNS WHO grade 3 tumors lived longer than patients with CNS WHO grade 4 tumors (*p* = 0.023) (Table S1, Fig. 1b). Since *CDKN2A* loss is a defining feature of CNS WHO grade 4 and since there was insufficient tissue to complete *CDKN2A* assessment for all CNS WHO grade 2 and 3 tumors, we performed a sensitivity analysis omitting all CNS WHO grade 2 and 3 tumors without *CDKN2A* assessment. These analyses revealed essentially the same survival curves (Fig. S1a, b), confirming that *CDKN2A* loss is infrequent in morphologically defined CNS WHO grade 2 and 3 tumors.

### Molecular marker profiles and outcome: type of IDH mutation and *MGMT* promoter methylation

Figure S2 shows survival curves stratified by IDH mutation type, i.e., IDH1-R132H versus all other (non-canonical) *IDH1* or *IDH2* mutations. In the entire cohort (Fig. S2a) as well as in the grade 2 (Fig. S2b) and grade 4 (Fig. S2d) subcohorts, there were no differences in OS by IDH mutation type. Only in patients with CNS WHO grade 3 tumors, presence of a non-canonical IDH mutation was associated with a better outcome (*p* = 0.021) (Fig. S2c).

We also compared the outcome by *MGMT* promoter methylation status across the entire cohort and by CNS WHO grade. In the entire cohort, *MGMT* promoter methylation was not prognostic for PFS but inversely related to OS (Fig. S3a,b). The latter finding is explained by the overall lower frequency of *MGMT* promoter methylation in the CNS WHO grade 2 tumors compared with CNS WHO grade 3 and 4 tumors (Table 1). Among patients with CNS WHO grade 4 tumors, *MGMT* promoter methylation was associated with longer PFS, but not OS (Fig. S3c, d, Table S2). Within the cohorts of patients with CNS WHO grade 2 or 3 tumors, the *MGMT* promoter methylation status was not related to PFS and OS (Fig. S3e–h). *MGMT* promoter methylation status was also not related to OS when survival analyses were restricted to patients exposed to alkylating agents (Fig. S4).

### Molecular markers associated with CNS WHO grade 4: *CDKN2A* homozygous deletion and lower levels of *LINE-1* methylation

Homozygous deletion of *CDKN2A* is per definition restricted to IDH-mutant astrocytomas of CNS WHO grade 4 [30] where it has been detected in 20–40% of tumors [26, 41]. We confirmed that homozygous *CDKN2A* deletion was highly prognostic over the complete dataset encompassing all CNS WHO grades (Fig. 1c,d) and remained prognostic among CNS WHO grade 4 tumors (Fig. 1e,f).

**Fig. 2:**
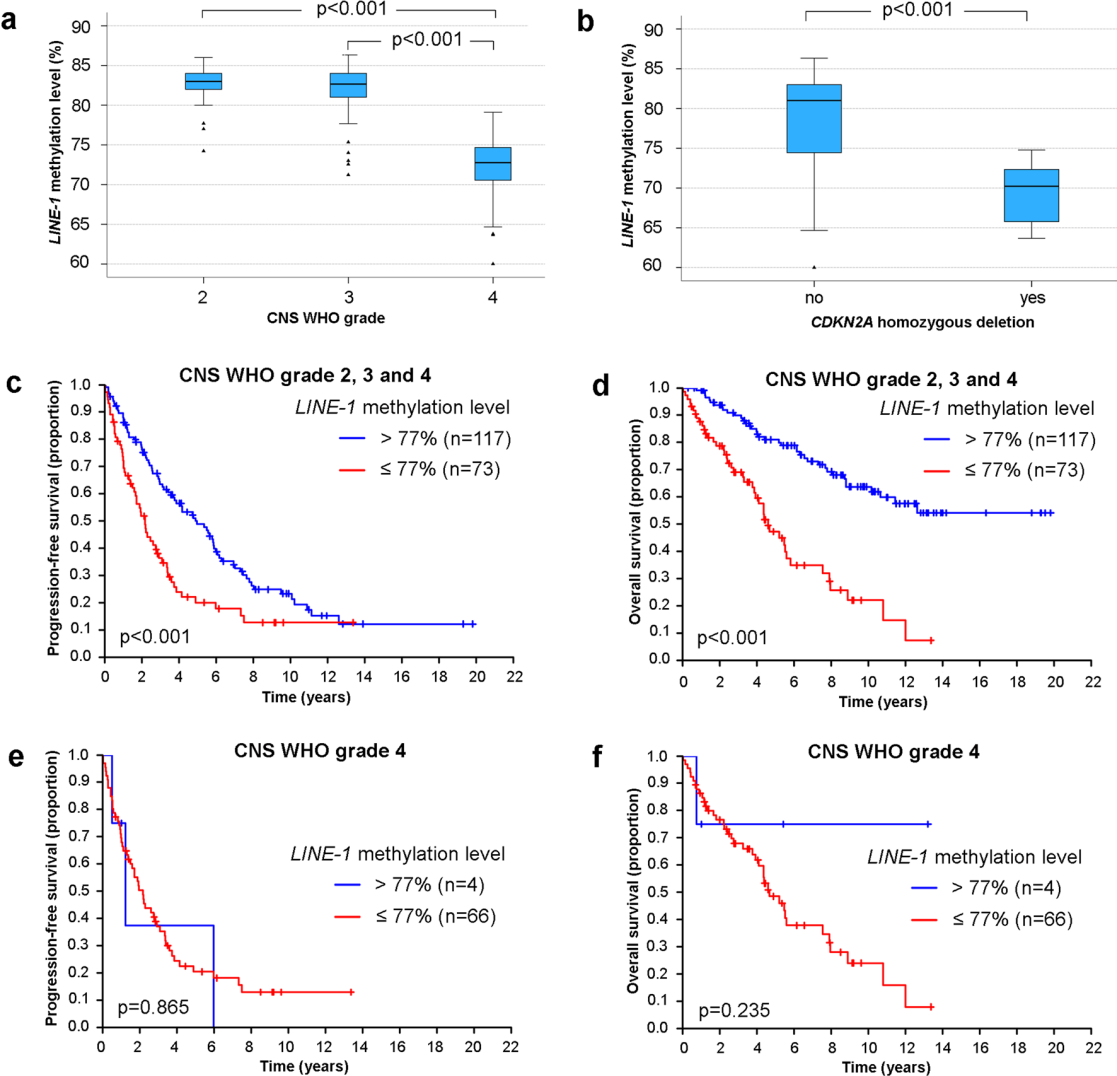
*LINE-1* methylation levels in IDH-mutant astrocytomas. *LINE-1* methylation levels according to CNS WHO grade 2 (*n* = 62), CNS WHO grade 3 (*n* = 58), and CNS WHO grade 4 (*n* = 70) (**a**). *LINE-1* methylation levels according to *CDKN2A* deletion status based on 11 tumors with homozygous *CDKN2A* deletion and 178 tumors without this alteration (**b**). PFS (**c**) and OS (**d**) of the entire patient cohort according to *LINE-1* methylation level stratified into ≤77% methylated alleles versus >77% methylated alleles. PFS (**e**) and OS (**f**) of the patients with CNS WHO grade 4 tumors according to *LINE-1* methylation levels

*LINE-1* methylation levels were significantly lower in CNS WHO grade 4 compared to lower grade tumors (Fig. 2a). IDH-mutant astrocytomas with homozygous *CDKN2A* deletion showed lower *LINE-1* methylation levels than IDH-mutant astrocytomas without complete *CDKN2A* loss (Fig. 2b). Correlation of *LINE-1* methylation levels with survival using a cut-off of 77% methylated alleles revealed that patients whose tumors had *LINE-1* methylation levels of ≤ 77% showed less favorable PFS and OS (Fig. 2c,d). Among patients with CNS WHO grade 4 tumors, *LINE-1* methylation levels ≤77% were not associated with shorter PFS or OS, a finding likely related to the low fraction of tumors with *LINE-1* methylation level >77% (*n* = 4) (Fig. 2e,f). We also performed a comparative analysis between *LINE-1* methylation levels and the assignment of tumors into the methylation classes “astrocytoma, IDH-mutant, lower grade” or “astrocytoma, IDH-mutant, high-grade” according to the Heidelberg brain tumor classified v.12b5 (www.molecularneuropathology.org) using available 450 k DNA methylome data of 85 IDH-mutant astrocytomas included in our cohort, with *LINE-1* methylation data being available for 80 of these cases. Overall, 31 of 31 CNS WHO grade 2 and 29 of 31 CNS WHO grade 3 tumors were assigned to the methylation class “astrocytoma, IDH-mutant, lower grade”, while 17 of 23 CNS WHO grade 4 tumors were assigned to the methylation class “astrocytoma, IDH-mutant, high-grade”. Similar to lower levels of *LINE*-*1* methylation, the DNA methylation class “astrocytoma, IDH-mutant, high-grade” was associated with CNS WHO grade 4 (*p* < 0.001). Correspondingly, *LINE-1* methylation levels were significantly lower in tumors assigned to the methylation class “astrocytoma, IDH-mutant, high-grade” (Fig. S5a). The previously determined *LINE-1* cut-off value of ≤77% methylated alleles was applied to assess its value to discriminate the two methylation classes. Overall, sensitivity for detection of the “astrocytoma, IDH mutant, high grade” methylation class by a *LINE-1* methylation level of ≤77% was 94.7% (95% CI 74.0–99.9%) and specificity was 90.2% (95% CI 79.8–96.3%). Among 23 CNS WHO grade 4 tumors with available 450 k DNA methylome data, a similar trend of lower *LINE-1* methylation levels in “astrocytoma, IDH-mutant, high-grade” tumors was observed, albeit the *p *value remained insignificant likely due to the low number of tumors assigned to the methylation class “astrocytoma, IDH-mutant, lower grade” among the CNS WHO grade 4 tumors (Fig. S5b).

### Prognostic factor analyses

Univariate analyses over the entire cohort revealed that CNS WHO grade, age, KPS, extent of resection, *CDKN*2A deletion, and *LINE*-*1* methylation level were prognostic. *MGMT* promoter methylation was prognostic, too, but with a better outcome for patients with tumors lacking *MGMT* promoter methylation (see above). On multivariate analysis, CNS WHO grade, extent of resection, and *CDKN*2A deletion were retained as prognostic factors (Table 2). Similar results were obtained when the same analyses were restricted to patients with IDH-mutant astrocytoma, CNS WHO grade 4 (Table S3).

**Table 2:** Prognostic factors in IDH-mutant astrocytoma: univariate and multivariate analyses

Entire cohort, univariate analyses	Hazard ratio	*p *value	95% CI
CNS WHO grade			
2 (ref)	–		
3	2.35	<0.001	1.47–3.76
4	4.14	<0.001	2.60–6.57
Age (years)			
> 40 versus ≤ 40 (ref.)	1.61	0.013	1.10–2.34
KPS			
<80 versus ≥80 (ref.)	2.55	<0.001	1.53–4.25
Surgery			
No total versus total (ref.)	1.73	0.017	1.10–2.73
*MGMT* promoter status			
Methylated versus unmethylated (ref.)	1.64	0.043	1.01–2.64
IDH mutation status			
IDH1-R132H versus other *IDH1* or *IDH2* mutations (ref.)	1.66	0.169	0.81–3.40
*CDKN2A* deletion status			
Homozygous versus no homozygous (ref.)	6.04	<0.001	2.90–12.56
*LINE-1* methylation status			
≤77 versus >77% methylated alleles (ref.)	3.54	<0.001	2.25–5.56

Entire cohort, multivariate analyses	Hazard ratio	*p* value	95% CI
CNS WHO grade			
2 (ref)	–		
3	3.09	0.007	1.36–7.00
4	1.42	0.577	0.42–4.80
Age (years)			
> 40 versus ≤ 40 (ref.)	1.58	0.098	0.92–2.72
KPS			
<80 versus ≥80 (ref.)	1.17	0.627	0.621–2.21
Surgery			
No total versus total (ref.)	2.61	< 0.001	1.49–4.58
*MGMT* promoter status			
Methylated versus unmethylated (ref.)	1.03	0.936	0.54–1.96
IDH mutations status			
IDH1-R132H versus other *IDH1* or *IDH2* mutations (ref.)	1.85	0.165	0.78–4.39
*CDKN2A* deletion status			
Homozygous versus no homozygous (ref.)	3.74	0.008	1.41–9.92
*LINE-1* methylation status			
≤77 versus >77% methylated alleles (ref.)	3.39	0.025	1.17–9.85

### Combination of CNS WHO grade and molecular biomarkers for improved prediction of outcome

**Fig. 3:**
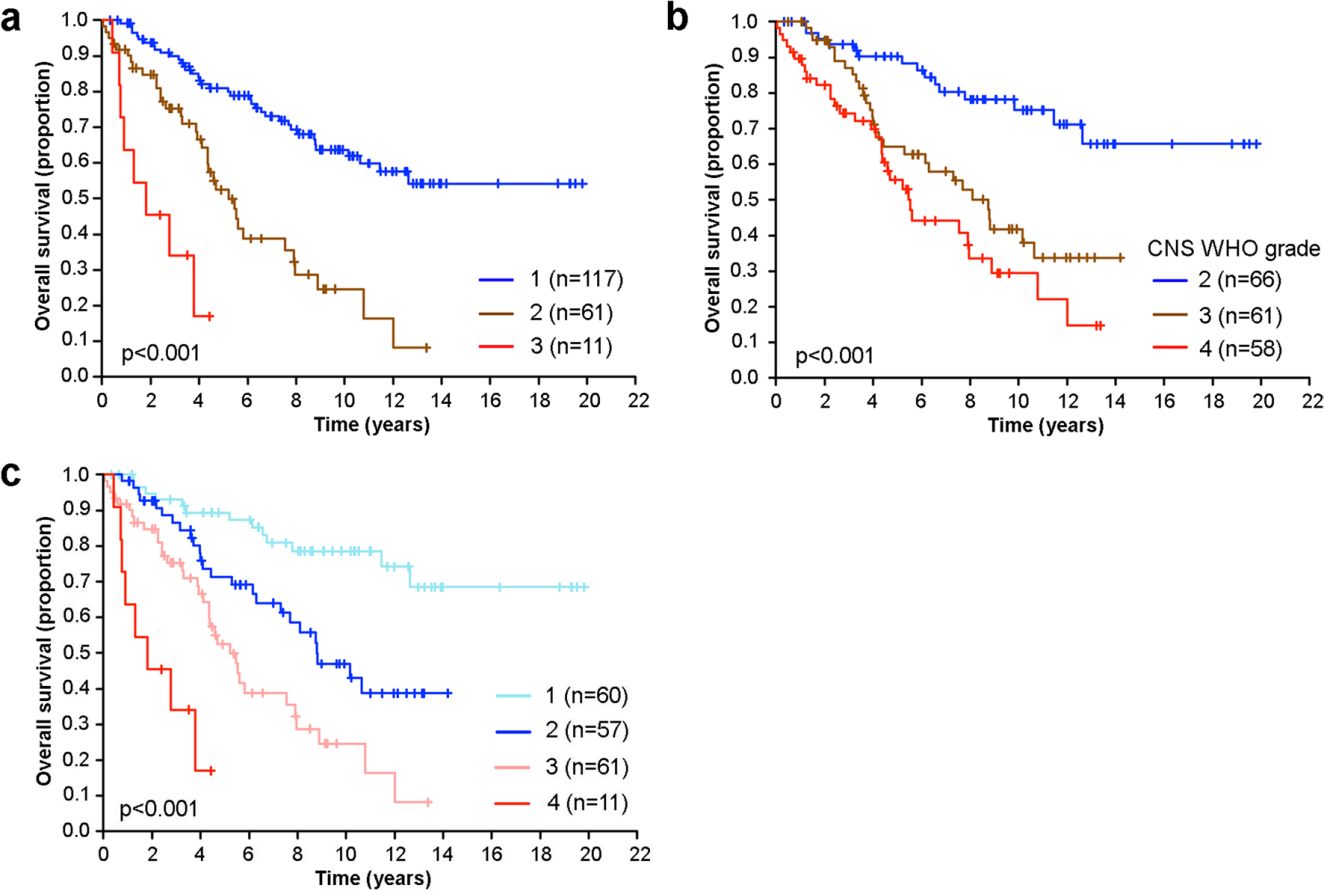
Prognostic stratification based on CNS WHO grade, *LINE-1* methylation level, and *CDKN2A* homozygous deletion status. (**a**) OS stratified by *LINE-1* methylation level (77% cut-off) and *CDKN2A* homozygous deletion status. The three distinct groups of patients correspond to: 1, *LINE-1* methylation levels of >77% without homozygous *CDKN2A* deletion; 2, *LINE-1* methylation levels of ≤77% without homozygous *CDKN2A* deletion; 3, *LINE-1* methylation levels of ≤77% with homozygous *CDKN2A* deletion). (**b**) OS by CNS WHO grade with omission of patients with *CDKN2A* homozygously deleted tumors. (**c**) OS stratified based on *LINE-1* methylation levels followed by CNS WHO grade 2 versus CNS WHO grade 3 or 4 in tumors with high *LINE-1* methylation levels or followed by *CDKN2A* homozygous deletion status in tumors with low *LINE-1* methylation levels The four distinct groups of patients correspond to: 1, *LINE-1* methylation levels of >77% and CNS WHO grade 2; 2, *LINE-1* methylation levels of >77% and CNS WHO grade 3 or 4; 3, *LINE-1* methylation levels of ≤77% without homozygous *CDKN2A* deletion; and 4, *LINE-1* methylation levels of ≤77% and homozygous *CDKN2A* deletion

Next we explored whether our findings might provide a new approach for improved prognostic assessment of patients with IDH-mutant astrocytoma. Stratification according to *LINE-1* methylation levels and *CDKN2A* copy number status resulted in three groups of patients with distinct overall survival (Fig. 3a). Group assignment remained highly significant upon adjustment for other prognostic factors (Table S4). Since *CDKN2A* homozygous deletion was a profound negative prognostic factor in our cohort (Figs. 1c–f), we also explored the prognostic significance of the current WHO classification when patients with *CDKN2A* homozygously deleted tumors were excluded. This resulted in a less distinct separation of outcome of CNS WHO grade 3 and 4 tumors (Fig. 3b). Yet, in tumors without homozygous *CDKN2A* deletion, lower *LINE-1* methylation levels were highly associated with CNS WHO grade 4 (Figs. S6a, b). Finally, placing *LINE-1* methylation levels at the apex of the prognostic stratification delineated four subgroups with relevant significant outcome differences (group 2 versus 1, HR = 2.99, *p* = 0.011; group 3 versus 2, HR = 1.68, *p* = 0.095; group 4 versus 3, HR = 3.51, *p* = 0.010).

In the group of patients whose tumors showed high *LINE-1* methylation levels (>77% methylated alleles), outcome was significantly different for patients with CNS WHO grade 2 tumors versus patients with CNS WHO grade 3 or (rare) grade 4 tumors. In the group of patients whose tumors showed low *LINE-1* methylation levels (≤77%), outcome was significantly different for patients with *CDKN2A* homozygously deleted tumors versus patients whose tumors had no homozygous *CDKN2A* deletion (Fig. 3c). The differences in outcome between the four groups were significant on univariate and multivariate analysis except for group 3 versus 2 (Table S5).

## Discussion

The present study provides contemporary data on the patterns of presentation, treatment, and outcome in the newly defined adult-type diffuse glioma group of IDH-mutant astrocytomas [7, 30] with a particular focus on astrocytoma, IDH-mutant, CNS WHO grade 4. The grading of IDH-mutant astrocytomas remains a matter of ongoing controversy [7, 18, 30, 34, 38]. Here, we report that stratification of these tumors into CNS WHO grades 2, 3, or 4 as recently defined in the WHO 2021 classification [30] is prognostically important (Fig. 1). Currently, the distinction of CNS WHO grade 2 versus 3 according to the WHO classification 2021 relies on the presence of focal or dispersed anaplasia and significant mitotic activity; however, a distinct cut-off for the mitotic count was not established [30]. A recent study based on patients included in the EORTC trials 26053 (CATNON) and 22033–26033 supported a prognostic role of mitotic activity and reported that a cut-off of two mitoses per ten microscopic high power fields was linked to significantly longer PFS and marginally longer OS in patients with IDH-mutant astrocytoma without homozygous *CDKN2A/CDKN2B* deletion [28]. Another recent study reported that the combination of <6 mitoses per 3 mm^2^ and a residual tumor volume of <1 cm^2^ upon postsurgical imaging was indicative of longer time to treatment and overall survival in patients with IDH-mutant astrocytomas of CNS WHO grade 2 or 3 [45].

The majority of IDH-mutant astrocytomas, CNS WHO grade 4, present de novo, rather than arising from a pre-existing lower grade astrocytoma [26]. The similar age at diagnosis across the groups defined by CNS WHO grade reported here (Table 1) supports this notion. A recent analysis of pooled data from clinical trials suggested that the canonical R132H mutation may confer an inferior survival compared with the less common, non-canonical mutations in *IDH1* or mutations in *IDH2* [44]. This association was confirmed in a cohort from Italy [17] and a recent meta-analysis [12], while data from the French POLA cohort revealed no clear prognostic association by type of IDH mutation [35]. We observed an association of non-canonical IDH mutations with longer OS only in the group of patients with CNS WHO grade 3 tumors (Fig. S2), but the sample size was overall small.

The limited prognostic role of *MGMT* promoter methylation status despite the broad use of alkylating agents in our patient population was surprising, but may confirm a recent cohort study [25] and is in line with previous data indicating *MGMT* promoter methylation as a predictive marker of response to alkylating agents in IDH-wildtype glioblastoma but not in IDH-mutant gliomas [53]. We observed *MGMT* promoter methylation in IDH-mutant astrocytomas of CNS WHO grade 2 less frequently than in CNS WHO grade 3 or 4 tumors, a finding which might contribute to the lack of prognostic association of the *MGMT* status.

Our study confirms the strong negative prognostic value of *CDKN2A* homozygous deletion in IDH-mutant astrocytoma patients. As reported before [16, 26, 41], presence of a *CDKN2A/CDKN2B* homozygous deletion is associated with particularly poor outcome of IDH-mutant astrocytoma patients, even within the group of patients with CNS WHO grade 4 tumors [26]. Thus, our findings lend further support for this molecular alteration as an independent indicator of CNS WHO grade 4 behavior [30]. The WHO classification recommends diagnostic testing for *CDKN2A/CDKN2B* homozygous in IDH-mutant astrocytomas showing histological features of anaplasia corresponding to CNS WHO grade 3, but not for IDH-mutant astrocytomas with histological features corresponding to CNS WHO grade 2 tumors [30], as the latter generally lack *CDKN2A/CDKN2B* homozygous deletion [41] (Table 1). However, *CDKN2A/CDKN2B* homozygous deletion is not very common even in CNS WHO grade 4 tumors, and novel markers of CNS WHO grade 4 that can be easily tested in clinical practice are urgently needed.

Here, we report that the *LINE-1* methylation level, a surrogate marker for the global DNA methylation status, is markedly lower in IDH-mutant astrocytomas of CNS WHO grade 4 compared with lower-grade tumors. So far, *LINE-1* methylation levels have not been studied in depth in gliomas. One study reported lower *LINE-1* methylation levels in glioblastomas compared to low-grade gliomas, and higher *LINE-1* methylation levels were associated with *MGMT* promoter methylation and longer survival of glioblastoma patients [33]. Another study revealed that high levels of *LINE-1* methylation and gene-specific hypermethylation of several genes were linked to longer survival of glioma patients [59]. However, both studies were based on histologically classified glioblastomas and lower grade diffuse gliomas, i.e., did not stratify the investigated cohorts according to the IDH mutation status. Here, we found a significantly lower level of *LINE-1* methylation in IDH-mutant astrocytomas of CNS WHO grade 4 compared with IDH-mutant astrocytomas of CNS WHO grade 2 or 3. In addition, lower *LINE-1* methylation levels were associated with shorter survival in IDH-mutant astrocytoma patients. Our findings confirm large-scale methylome analyses that identified a subset of IDH-mutant astrocytomas with lower levels of global DNA methylation and shorter survival, which were referred to as “glioma-CpG island methylator phenotype (G-CIMP)-low” tumors as opposed to “G-CIMP-high” tumors [11, 31, 42, 43]. Along this line, array-based DNA methylome profiling using the Heidelberg methylation classifier version v.12.5 identifies two distinct DNA methylation classes of IDH-mutant astrocytoma, namely “astrocytoma, IDH-mutant, lower grade” and “astrocytoma, IDH-mutant, high-grade” (www.molecularneuropathology.org) [9], which largely overlap with the “G-CIMP-high” and “G-CIMPlow” groups, respectively [42]. Correlative analysis in relation to these two distinct methylation classes consequently revealed lower levels of *LINE-1* methylation in the “astrocytoma, IDH-mutant, high-grade” methylation class. Taken together, our findings, thus, confirm lower levels of global DNA methylation as a prognostically unfavorable molecular alteration in IDH-mutant astrocytomas [42, 43, 56] that can be detected by DNA methylation arrays and other methods like *LINE-1* methylation analysis using pyrosequencing, i.e., a method already established in many laboratories for the assessment of the *MGMT* promoter methylation status [54]. Hence, detection of lower levels of *LINE-1* methylation may represent a novel biomarker that may support grading of IDH-mutant astrocytoma by indicating CNS WHO grade 4 behavior. In our study, a *LINE-1* methylation level of 77% calculated across three selected *LINE-1* CpG sites was employed to distinguish high level versus low-level methylated cases. However, quantitative methylation levels may vary according to specific assays and equipment used; hence, validation and potential adaptation of cut-offs to work-flows used in other laboratories will likely be required, as also indicated by the variable levels of *LINE-1* methylation reported in astrocytic gliomas before [33, 59].

*LINE-1* methylation levels were an independent prognostic variable for survival upon multivariate analysis (Table 2). However, individual cases of CNS WHO grade 2 and 3 tumors showed *LINE-1* methylation levels of ≤77% while individual cases of CNS WHO grade 4 tumors without *CDKN2A* homozygous deletion had *LINE-1* methylation levels of >77% (Fig. S6b). In addition, other authors reported on a prognostically unfavorable association of lower global DNA methylation levels in a cohort of IDH-mutant grade 4 astrocytic gliomas/glioblastomas [56]. Thus, determination of the global DNA methylation level may provide information beyond WHO grading, as supported by other studies [11, 31].

Limitations of our study include the retrospective design, potential bias of enrolling patients with favorable outcome into cohorts like the GGN and lack of standardized approaches to treatment and follow-up. We also did not quantitatively evaluate mitotic count as performed in the recent studies by Kros et al. [28] and Tran et al. [45] that reported on various cut-offs for mitotic counts predicting outcome. In addition, the *LINE-1* cut-off used in this study would require independent validation in a distinct patient cohort. Furthermore, potential diagnostic use in individual patients would demand the establishment of a validated assay that is standardized concerning, among others, the definition of CpG sites to be interrogated, the method for calculation of methylated allele frequencies, input amount of DNA and completeness of bisulfite conversion, appropriate control samples, and the actual pyrosequencing protocol. Nevertheless, the present cohort of patients with IDH-mutant astrocytomas is relatively large and may serve as a framework for further efforts aiming at characterizing novel markers for improved prediction of therapy response and outcome that could also guide treatment strategy and clinical trial design, notably with the view to defining the role of IDH inhibitors along the disease trajectory [32]. Furthermore, we provide possible future avenues to improve histomolecular prognostic assessment of IDH-mutant astrocytoma based on CNS WHO grade, global DNA methylation level, and *CDKN2A* homozygous deletion (Fig. 3).

### Supplementary Information

Below is the link to the electronic supplementary material.

Supplementary file1 (DOCX 20890 KB)

## Data Availability

Anonymized data may be shared upon appropriate request of qualified investigators for purposes of replicating procedures and results.

## Abbreviations

CI: Confidence interval
CNS: Central nervous system
GGN: German Glioma Network
HR: Hazard ratio
IDH: Isocitrate dehydrogenase
KPS: Karnofsky performance status
*LINE-1*: Long interspersed nuclear element 1
*MGMT*: O^6^-methylguanine-DNA methyltransferase
OS: Overall survival
PFS: Progression-free survival
RT: Radiotherapy
TMZ: Temozolomide
WHO: World Health Organization
