# Supplementary material for: Improved prognostic stratification of patients with isocitrate dehydrogenase-mutant astrocytoma
Source: Acta Neuropathol. 2024 Jan 6;147(1):11. doi: 10.1007/s00401-023-02662-1 (PMC10771615; doi:10.1007/s00401-023-02662-1)
Supplement: Supplementary file 1 — Supplementary file1 (DOCX 20890 KB) [file 401_2023_2662_MOESM1_ESM.docx]

**Improved prognostic stratification of patients with isocitrate dehydrogenase-mutant astrocytoma**

Michael Weller, Jörg Felsberg, Bettina Hentschel, Dorothee Gramatzki, Nadezhda Kubon, Marietta Wolter, Matthias Reusche, Patrick Roth, Dietmar Krex, Ulrich Herrlinger, Manfred Westphal, Joerg C. Tonn, Luca Regli, Claude-Alain Maurage, Andreas von Deimling, Torsten Pietsch, Emilie Le Rhun, Guido Reifenberger

**Corresponding author**

Michael Weller, MD, Department of Neurology, University Hospital Zurich, Frauenklinikstrasse 26, 8091 Zurich, Switzerland, Telephone +41 44 255 5500, E-mail: michael.weller@usz.ch

**Supplementary Tables and Figures**

**Supplementary Table S1.** Treatment and outcome by CNS WHO grade.

**Supplementary Table S2.** Outcome of patients with IDH-mutant astrocytoma by CNS WHO grade and *MGMT* promoter methylation status.

**Supplementary Table S3.** Prognostic factors in patients with IDH-mutant astrocytoma, CNS WHO grade 4: univariate and multivariate analyses.

**Supplementary Table S4.** Univariate and multivariate analyses of survival of IDH-mutant astrocytoma patients according to the three prognostic subgroups defined by *LINE-1* methylation level and *CDKN2A* status (Figure 3a).

**Supplementary Table S5.** Univariate and multivariate analyses of survival of IDH-mutant astrocytoma patients according to the four prognostic subgroups defined by *LINE-1* methylation level, CNS WHO grade and *CDKN2A* status (Figure 3b).

**Supplementary Figure S1.** Outcome of patients with IDH-mutant astrocytoma stratified according to CNS WHO grade.

**Supplementary Figure S2.** Outcome of patients with IDH-mutant astrocytoma stratified according to type of IDH mutation.

**Supplementary Figure S3.** Outcome of patients with IDH-mutant astrocytoma according to *MGMT* promoter methylation status.

**Supplementary Figure S4.** Outcome of patients with IDH-mutant astrocytoma, CNS WHO grade 2, 3 or 4 according to *MGMT* promoter methylation status, with documented alkylating agent chemotherapy.

**Supplementary Figure S5.** *LINE-1* methylation levels in relation to DNA methylation class determined by 450K DNA methylation arrays.

**Supplementary Figure S6.** *LINE-1* methylation levels in IDH-mutant astrocytomas without homozygous *CDKN2A* deletion (n=178) according to CNS WHO grade.

**Supplementary Table S1. Treatment and outcome by CNS WHO grade**.

|  | Astrocytoma,  IDH-mutant,  CNS WHO grade 2  n=114 | Astrocytoma,  IDH-mutant,  CNS WHO grade 3  n=73 | Astrocytoma,  IDH-mutant,  CNS WHO grade 4  n=71 |
| --- | --- | --- | --- |
| **Surgery (extent of resection)^a^** |  |  |  |
| Gross total resection | 27 (29.7%) | 18 (30.0%) | 29 (42.6%) |
| Subtotal resection | 25 (27.5%) | 22 (36.7%) | 23 (33.8%) |
| Partial resection | 16 (17.6%) | 14 (23.3%) | 9 (13.2%) |
| Biopsy | 23 (25.3%) | 6 (10.0%) | 7 (10.3%) |
| No data | 23 | 13 | 3 |
| **First-line treatment** |  |  |  |
| RT alone | 9 (7.9%) | 17 (23.3%) | 3 (4.2%) |
| TMZ/RT→TMZ | 2 (1.8%) | 16 (21.9%) | 51 (71.8%) |
| Maintenance TMZ cycles median (range) | n.a. | 6 (0-13) | 6 (0-71) |
| Patients with information on number of TMZ cycles | 1/2 | 15/16 | 47/51 |
| TMZ alone | 11 (9.6%) | 10 (13.7%) | 2 (2.8%) |
| TMZ/RT/bevacizumab | 0 | 0 | 3 (4.2%) |
| PC/PCV alone | 2 (1.8%) | 3 (4.1%) | 1 (1.4%) |
| RT/other | 2 (1.8%) | 13 (17.8%) | 3 (4.2%) |
| Other^b^ | 0 | 0 | 4 (5.6%) |
| No further therapy | 88 (77.2%) | 14 (19.2%) | 4 (5.6%) |
| **PD (events)** | 84 (73.7%) | 49 (67.1%) | 56 (78.9%) |
|  |  |  |  |
| **Management at first progression** | 84 (100%) | 49 (100%) | 56 (100%) |
| No salvage therapy | 8 (9.5%) | 9 (18.4%) | 10 (17.9%) |
| Surgery alone | 12 (14.3%) | 4 (8.2%) | 4 (7.1%) |
| Surgery plus RT | 9 (10.7%) | 3 (6.1%) | 2 (3.6%) |
| Surgery plus alkylating CT | 11 (13.1%) | 9 (18.4%) | 7 (12.5%) |
| Surgery plus RT plus alkylating CT | 17 (20.2%) | 1 (2.0%) | 4 (7.1%) |
| Surgery plus other treatment | 1 (1.2%) | 0 | 2 (3.6%) |
| RT alone | 11 (13.1%) | 4 (8.2%) | 2 (3.6%) |
| Alkylating CT alone | 10 (11.9%) | 13 (26.5) | 16 (28.6%) |
| RT plus alkylating CT | 4 (4.8%) | 6 (12.2%) | 3 (5.4%) |
| Other salvage treatment | 1 (1.2%) | 0 | 6 (10.7 %) |
| **CNS WHO grade at progression** |  |  |  |
| No change | 14 | 11 | 19 |
| 2 to 3 | 26 | n.a. | n.a. |
| 2 to 4 | 10 | n.a. | n.a. |
| 3 to 4 | n.a. | 4 | n.a. |
| No data | 0 | 2 | 0 |
| **Survival** |  |  |  |
| Median PFS (years, 95% CI) | 4.6 (2.8-6.3) | 4.2 (2.6-5.8) | 2.0 (1.4-2.5) |
| Median overall survival (years, 95% CI) | not reached | 8.1 (5.4-10.8) | 4.7 (3.4-6.0) |
| Survival at 1 year (%, 95% CI) | 99.1 (97.3-100) | 100 | 85.8 (77.8-93.8) |
| Survival at 2 years (%, 95% CI) | 96.4 (92.9-99.9) | 95.7 (90.8-100) | 76.7 (66.7-86.7) |
| Median follow-up in years (range) | 10.4 (0.3-19.8) | 9.7 (0.5-18.4) | 6.5 (0.7-13.4) |
| Alive at last follow-up (n, %) | 78 (68.4%) | 37 (50.7%) | 30 (42.3%) |

Abbreviations:

CI, confidence interval; PD, progressive disease; TMZ, temozolomide; TMZ/RT→TMZ, radiotherapy with concomitant and maintenance TMZ.

^a^extent of resection was captured based on early postoperative neuroimaging and assessed locally, not by central review

^b^TMZ/bevacizumab (N=1), TMZ/RT/nivolumab or placebo (N=1; NCT 02667587), TMZ/RT/fotemustine (N=1), radioimmunotherapy (N=1)

**Supplementary Table S2. Outcome of patients with IDH-mutant astrocytoma by CNS WHO grade and *MGMT* promoter methylation status.**

|  | **Progression-free survival in years** (median (95% CI) | **Overall survival in years** (median. (95% CI) |
| --- | --- | --- |
| **CNS WHO grade 2** |  |  |
| *MGMT* promoter methylated | 4.5 (2.2-6.7) | 12.6 (10.3-15.0) |
| *MGMT* promoter unmethylated | 4.6 (2.5-6.7) | - |
| **CNS WHO grade 3** |  |  |
| *MGMT* promoter methylated | 4.9 (3.2-6.6) | 7.3 (4.8-9.8) |
| *MGMT* promoter unmethylated | 3.9 (0.6-7.1) | - |
| **CNS WHO grade 4** |  |  |
| *MGMT* promoter methylated | 2.3 (1.6-3.0) | 4.7 (3.4-6.0) |
| *MGMT* promoter unmethylated | 1.2 (0.7-1.8) | 5.5 (2.2-8.7) |

**Supplementary Table S3. Prognostic factors in patients with IDH-mutant astrocytoma, CNS WHO grade 4: univariate and multivariate analyses.**

| **IDH-mutant astrocytoma CNS WHO grade 4, univariate analyses** | **Hazard ratio** | **p-value** | **95% CI** |
| --- | --- | --- | --- |
| **Age (years)**  >40 vs. ≤40 (ref.) | 1.44 | 0.273 | 0.75–2.78 |
| **KPS**  <80 vs. ≥80 (ref.) | 1.38 | 0.322 | 0.73–2.62 |
| **Surgery**  No total vs. total (ref.) | 1.52 | 0.216 | 0.79–2.93 |
| ***MGMT* promoter status**  Methylated vs. unmethylated (ref.) | 0.83 | 0.649 | 0.38–1.84 |
| **IDH mutations status**  IDH1-R132H vs. other *IDH1* or *IDH2* mutations (ref.) | 1.12 | 0.864 | 0.34–3.63 |
| ***CDKN2A* deletion status**  Homozygous vs. no homozygous (ref.) | 3.39 | 0.003 | 1.51–7.61 |
| ***LINE-1*** **methylation status**  ≤77% vs. >77% methylated alleles (ref.) | 3.17 | 0.260 | 0.43–23.54 |

| **IDH-mutant astrocytoma CNS WHO grade 4, multivariate analyses** | **Hazard ratio** | **p-value** | **95% CI** |
| --- | --- | --- | --- |
| **Age (years)**  >40 vs. ≤40 (ref.) | 1.52 | 0.296 | 0.70 – 3.30 |
| **KPS**  <80 vs. ≥80 (ref.) | 1.36 | 0.405 | 0.66-2.83 |
| **Surgery**  No total vs. total (ref.) | 2.19 | 0.044 | 1.02-4.70 |
| ***MGMT* promoter status**  Methylated vs. unmethylated (ref.) | 0.79 | 0.601 | 0.32–1.92 |
| **IDH mutations status**  IDH1-R132H vs. other *IDH1* or *IDH2* mutations (ref.) | 1.24 | 0.733 | 0.36–4.34 |
| ***CDKN2A* deletion status**  Homozygous vs. no homozygous (ref.) | 2.72 | 0.053 | 1.00–7.47 |
| ***LINE-1*** **methylation status**  ≤77% vs. >77% methylated alleles (ref.) | 3.20 | 0.270 | 0.40–25.39 |

Abbreviations: CI, confindence interval; KPS, Karnofsky perfomance status; ref, reference

**Supplementary Table S4. Univariate and multivariate analyses of survival of IDH-mutant astrocytoma patients according to the three prognostic subgroups defined by *LINE-1* methylation level and *CDKN2A* deletion status (Figure 3a).** Other prognostic variables included are age, Karnofsky performance status (KPS), extent of resection, *MGMT* promoter methylation status and type of IDH mutation.

| **Univariate analyses** | **Hazard ratio** | **p-value** | **95% CI** |
| --- | --- | --- | --- |
| **Group^a^**  2 vs. 1 (ref.)  3 vs. 1 (ref.) | 3.17  10.11 | <0.001  <0.001 | 2.00–5.08  4.46–22.89 |
| **Age (years)**  >40 vs. ≤40 (ref.) | 1.85 | 0.006 | 1.19–2.88 |
| **KPS**  <80 vs. ≥80 (ref.) | 2.32 | 0.002 | 1.35-4.01 |
| **Surgery**  No total vs. total (ref.) | 1.93 | 0.013 | 1.15-3.24 |
| ***MGMT* promoter status**  Methylated vs. unmethylated (ref.) | 1.44 | 0.194 | 0.83–2.48 |
| **IDH mutations status**  IDH1-R132H vs. other *IDH1* or *IDH2* mutations (ref.) | 1.85 | 0.148 | 0.80–4.25 |

| **Multivariate analyses** | **Hazard ratio** | **p-value** | **95% CI** |
| --- | --- | --- | --- |
| **Group^a^**  2 vs. 1 (ref.)  3 vs. 1 (ref.) | 2.57  9.02 | 0.001  <0.001 | 1.44–4.59  3.30–24.70 |
| **Age (years)**  >40 vs. ≤ 40 (ref.) | 1.70 | 0.054 | 0.99–2.91 |
| **KPS**  <80 vs. ≥80 (ref.) | 1.27 | 0.466 | 0.67–2.40 |
| **Surgery**  No total vs. gross total (ref.) | 2.50 | 0.001 | 1.43-4.38 |
| ***MGMT* promoter status**  Methylated vs. unmethylated (ref.) | 1.16 | 0.647 | 0.62–2.19 |
| **IDH mutation status**  IDH1-R132H vs. other *IDH1* or *IDH2* mutations (ref.) | 1.81 | 0.179 | 0.76–4.30 |

^a^1 = *LINE-1* methylation level > 77% & no *CDKN2A* homozygous deletion

2 = *LINE-1* methylation level ≤ 77% & no *CDKN2A* homozygous deletion

3 = *LINE-1* methylation level ≤ 77% & *CDKN2A* homozygous deletion

**Supplementary Table S5. Univariate and multivariate analyses of survival of IDH-mutant astrocytoma patients according to the four prognostic subgroups defined by *LINE-1* methylation level, CNS WHO grade and *CDKN2A* status (Figure 3c).** Other prognostic variables included are age, Karnofsky performance status (KPS) extent of resection, *MGMT* promoter methylation and type of IDH mutation.

| **Univariate analyses** | **Hazard ratio** | **p-value** | **95% CI** |
| --- | --- | --- | --- |
| **Group^a,b^**  2 vs. 1 (ref.)  3 vs. 1 (ref.)  4 vs. 1 (ref.) | 2.76  5.55  18.01 | 0.003  <0.001  <0.001 | 1.41–5.42  2.89–10.65  7.05–45.97 |
| **Age (years)**  >40 vs. ≤40 (ref.) | 1.85 | 0.006 | 1.19–2.88 |
| **KPS**  <80 vs. ≥80 (ref.) | 2.32 | 0.002 | 1.35-4.01 |
| **Surgery**  No total vs. total (ref.) | 1.93 | 0.013 | 1.15-3.24 |
| ***MGMT* promoter status**  Methylated vs. unmethylated (ref.) | 1.44 | 0.194 | 0.83–2.48 |
| **IDH mutations status**  IDH1-R132H vs. other *IDH1* or *IDH2* mutations (ref.) | 1.85 | 0.148 | 0.80–4.25 |

| **Multivariate analyses** | **Hazard**  **ratio** | **p-value** | **95% CI** |
| --- | --- | --- | --- |
| **Group^a,c^**  2 vs. 1 (ref.)  3 vs. 1 (ref.)  4 vs. 1 (ref.) | 2.99  5.04  17.67 | 0.011  <0.001  <0.001 | 1.29–6.97  2.19–11.60  5.48–57.01 |
| **Age (years)**  > 40 vs. ≤ 40 (ref.) | 1.68 | 0.059 | 0.98–2.88 |
| **KPS**  < 80 vs. ≥80 (ref.) | 1.16 | 0.646 | 0.62–2.17 |
| **Surgery**  No total vs. total (ref.) | 2.56 | 0.001 | 1.46-4.49 |
| ***MGMT* promoter status**  Methylated vs. unmethylated (ref.) | 1.05 | 0.875 | 0.56–1.99 |
| **IDH mutations status**  IDH1-R132H vs. other *IDH1* or *IDH2* mutations (ref.) | 1.82 | 0.176 | 0.77–4.31 |

^a^1 = *LINE-1* methylation level > 77% & CNS WHO grade 2

2 = *LINE-1* methylation level > 77% & CNS WHO grade 3/4

3 = *LINE-1* methylation level ≤ 77% & no *CDKN2A* homozygous deletion

4 = *LINE-1* methylation level ≤ 77% & *CDKN2A* homozygous deletion

^b^Group 3 vs. 2 (ref.): HR=2.01, p=0.009, 95% CI 1.19-3.38

Group 4 vs. 3 (ref.): HR=3.25, p=0.004, 95% CI 1.47-7.19

^c^Group 3 vs. 2 (ref.): HR=1.68, p=0.095, 95% CI 0.91-3.10

Group 4 vs. 3 (ref.): HR=3.51, p=0.010, 95% CI 1.35-9.16

**Supplementary Figure S1. Outcome of patients with IDH-mutant astrocytoma stratified according to CNS WHO grade.** PFS (a) and OS (b) of the subgroup of patients with IDH-mutant astrocytoma and information on *CDKN2A* copy number status stratified by CNS WHO grade 2, 3 or 4.


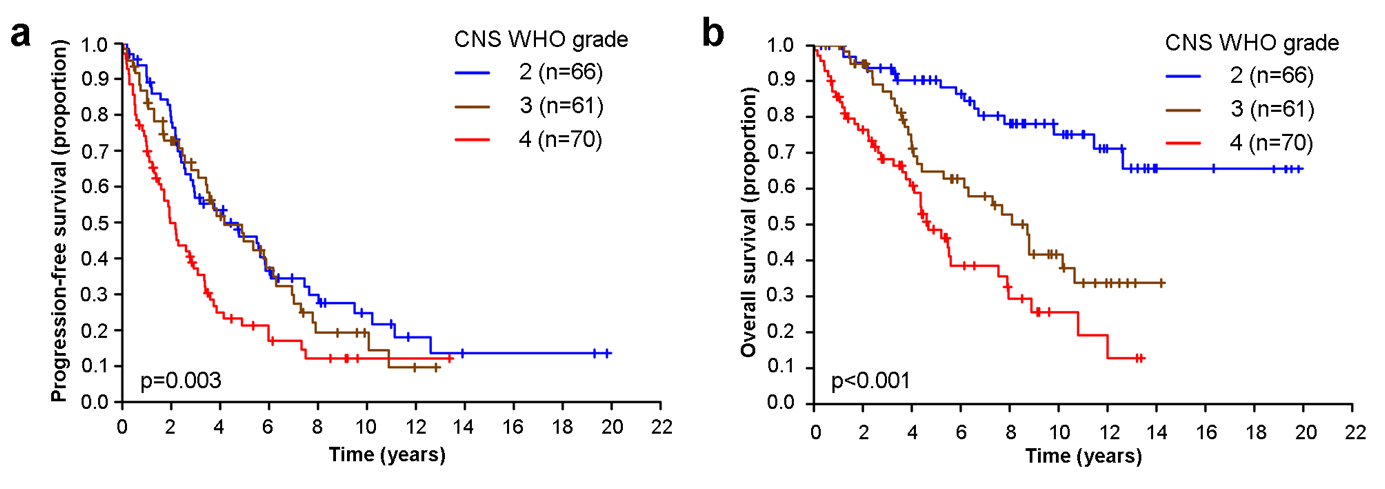


**Supplementary Figure S2. Outcome of patients with IDH-mutant astrocytoma stratified according to type of IDH mutation.** OS by canonical versus non-canonical IDH mutation in the entire cohort of patients (a) and in the patients with CNS WHO grade 2 (b), 3 (c) or 4 (d) tumors.


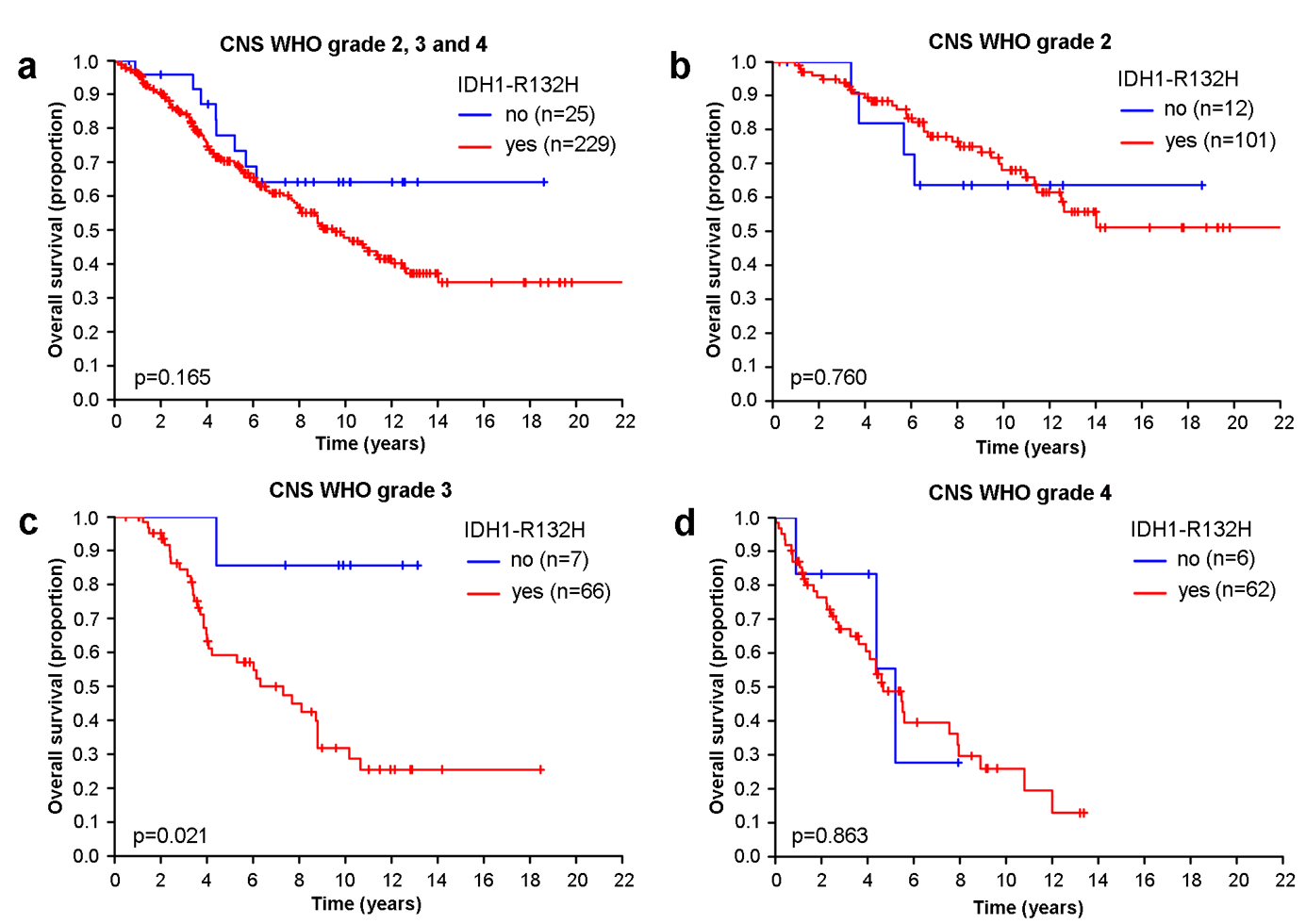


**Supplementary Figure S3. Outcome of patients with IDH-mutant astrocytoma according to *MGMT* promoter methylation status.** Kaplan-Meier survival curves illustrating PFS (a,c,e,g) and OS (b,d,f,h) according to *MGMT* promoter methylation status in the entire cohort of patients (a,b), the patients with CNS WHO grade 4 tumors (c,d), the patients with CNS WHO grade 3 tumors (e,f), or the patients with CNS WHO grade 2 tumors (g,h).


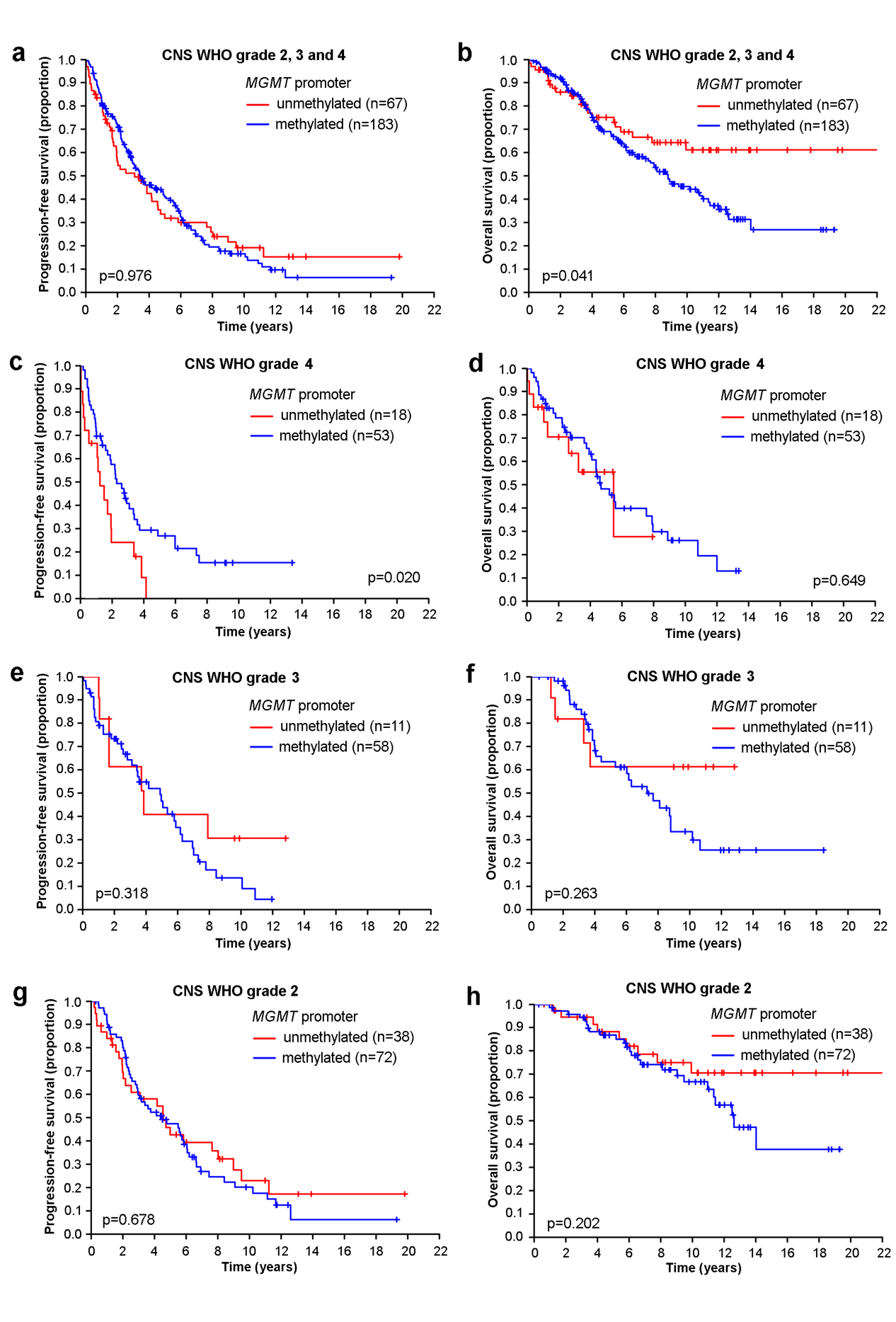


**Supplementary Figure S4.** **Outcome of patients with IDH-mutant astrocytoma, CNS WHO grade 2, 3 or 4 according to *MGMT* promoter methylation status, with documented alkylating agent chemotherapy.** Kaplan-Meier survival curves illustrating OS according to *MGMT* promoter methylation status in patients with IDH-mutant astrocytoma, CNS WHO grade 2 (a), 3 (b) or 4 (c).


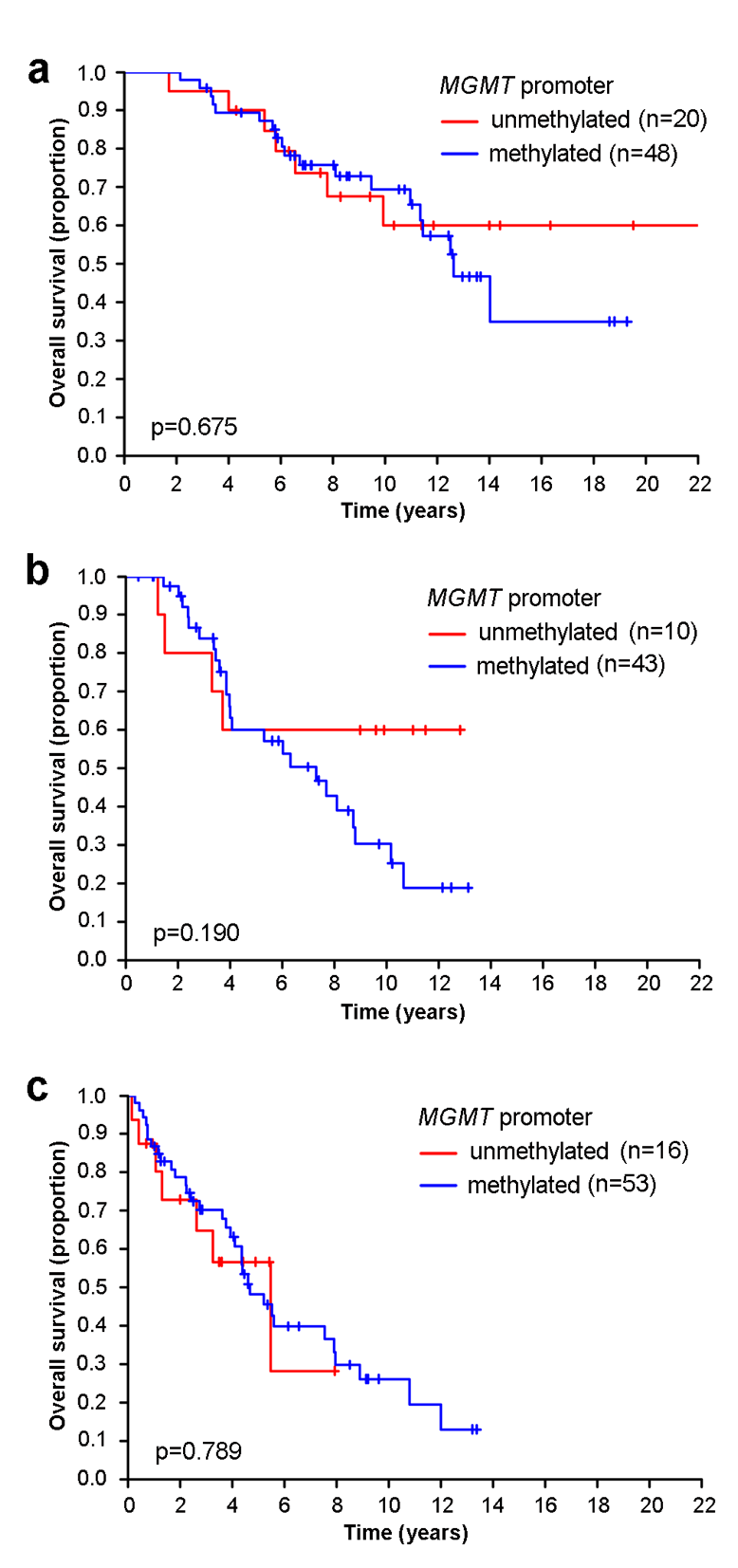


**Supplementary Figure S5. *LINE-1* methylation levels in relation to DNA methylation class determined by 450K DNA methylation arrays.** DNA methylation array data were available from 80 patients also tested for *LINE-1* methylation status in the current study. The tumors were assigned to the methylation classes "astrocytoma, IDH-mutant, lower grade (A IDHmut, lower grade)" or "astrocytoma, IDH-mutant, high-grade (A IDHmut, high-grade)" using the Heidelberg brain tumor classifier version v.12b5 (www.molecularneurpathology.org). In the entire group of patients, *LINE-1* methylation levels were significantly lower in the methylation class "A IDHmut, high-grade" (a). A similar trend was observed for the subset of CNS WHO grade 4 tumors, however, the difference here did not reach statistical significance, likely due to the small fraction of tumors (n=6) assigned to the methylation class "A IDHmut, lower grade" (b).


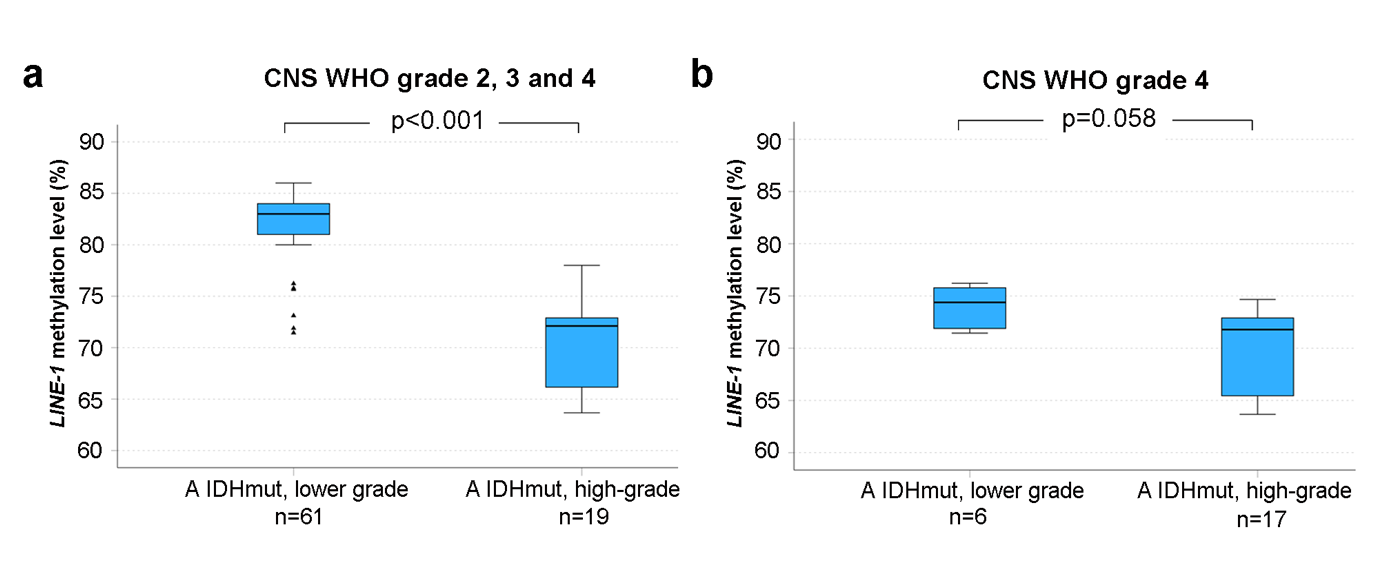


**Supplementary Figure S6. *LINE-1* methylation levels in IDH-mutant astrocytomas without homozygous *CDKN2A* deletion (n=178) according to CNS WHO grade.** Note that reduced global DNA methylation as indicated by LINE-1 methylation levels of ≤ 77% are significantly associated with CNS WHO grade 4 tumors (a). However, individual cases of CNS WHO grade 2 (n=2) or grade 3 (n=5) tumors displayed reduced *LINE-1* levels of ≤ 77% while a few CNS WHO grade 4 tumors (n=4) had *LINE-1* levels of > 77% (b).

**
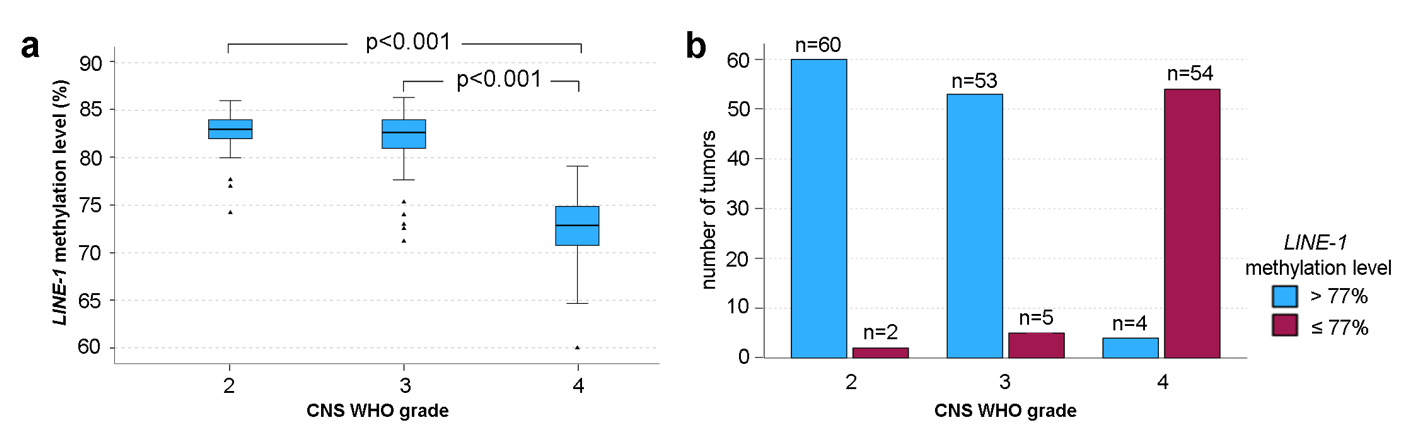
**
